# Extra Pulmonary Tuberculosis Manifesting as Liver Abscess

**DOI:** 10.7759/cureus.70988

**Published:** 2024-10-07

**Authors:** Settipally Vinay Kumar, Narayanasamy Senthil, Vaasanthi Rajendran, Nanthakumar Logithasan, Avinash Chenguttuvan

**Affiliations:** 1 General Medicine, Sri Ramachandra Institute of Higher Education and Research, Chennai, IND

**Keywords:** extrapulmonary tuberculosis, genexpert in hepatic abscess, hepatic tuberculosis, isolated tubercular liver abscess, mixed infection pyogenic and tubercular liver abscess

## Abstract

Liver abscess is a rare presentation of extrapulmonary tuberculosis. It may present with nonspecific symptoms such as fever, weight loss and abdominal pain and requires a high degree of suspicion. We present a case of a 57-year-old male previously treated for a liver abscess and presented with abdominal pain, vomiting and fever and a contrast-enhanced computed tomography (CECT) showed a loculated liver abscess. Ultrasound-guided aspiration of the pus was sent for GeneXpert MTB (Cepheid, Sunnyvale, CA, USA) and mycobacterial culture which confirmed the diagnosis. The patient was started on anti-tubercular therapy and clinically improved.

## Introduction

Hepatic tuberculosis (TB) can manifest in different ways as a part of miliary TB, and usually does not present with symptoms or signs pertaining to the liver [1,2]. Tuberculous hepatitis may present with fever, jaundice, and hepatomegaly [3]. Localized hepatic TB may present such as multiple nodules, tuberculoma, or hepatic abscess. Tubercular liver abscess is an uncommon manifestation of extrapulmonary TB, found in only 0.34% of cases [1].

## Case presentation

An adult male nonsmoker in his late 50s with a history of diabetes, alcoholism for the last 25 years with abstinence for the last eight months, and treatment with intravenous antibiotics for a liver abscess three months prior, presented to our department with vomiting and abdominal pain. He had a history of fever 10 days prior, which lasted two days and was resolved with antipyretics. He had no history of loose stools, loss of weight, travel, prior tubercular infection, or contact with anyone infected with TB. On examination, the patient was conscious with a systolic blood pressure of 80 mmHg and right hypochondrial tenderness. He was started on inotropes noradrenaline and vasopressin. Baseline investigations showed leukocytosis and deranged liver function tests (Table 1).

**Table 1:** Baseline laboratory investigations

Laboratory parameters	Patient’s value	Reference range
Haemoglobin	11.3 g/dL	12–17 g/dL
Total leukocyte count	14060/mm^3^	4000–11,000/mm^3^
Platelet count	268000/mm^3^	150,000–450,000/mm^3^
Erythrocyte sedimentation rate	91 mm/h	0–15 mm/h
Serum creatinine	0.7 mg/dL	0.8–1.3 mg/dL
Total bilirubin	2.86 mg/dL	0.3–1.2 mg/dL
Direct bilirubin	1.7 mg/dL	<0.2 mg/dL
Albumin	3.3 g/dL	3.5–5.2 g/dL
Serum aspartate aminotransferase (AST)	301 IU/L	<50 IU/L
Serum alanine aminotransferase (ALT)	171 IU/L	<50 IU/L
Serum alkaline phosphatase (ALP)	652 IU/L	32–120 IU/L
Gamma-glutamyl transferase	256 IU/L	5–40 IU/L
Serum sodium	129 meq/L	136–146 meq/L
Serum potassium	3.1 meq/L	3.5–5.1 meq/L
Serum chloride	97 meq/L	101–109 meq/L
Serum bicarbonate	19 meq/L	21–31 meq/L

**Figure 1:**
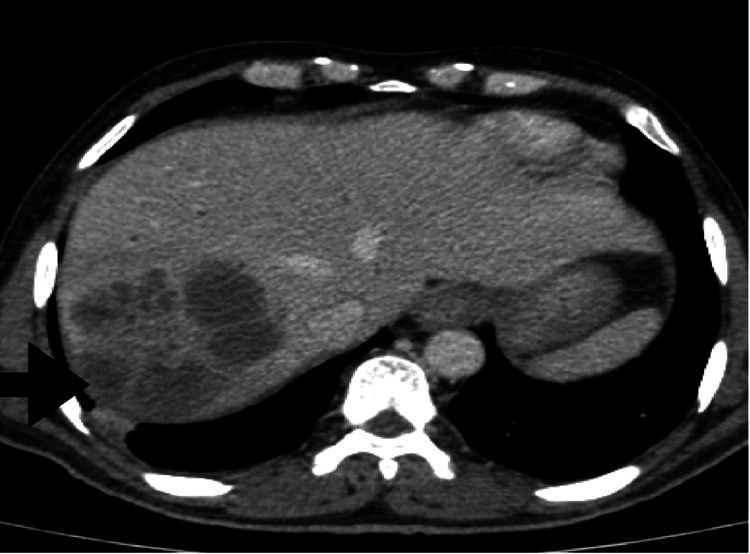
Contrast-enhanced computed tomography of the abdomen showing multiloculated hypodense lesion with multiple internal septations measuring 6.9 x 7.4 x 7.5 cm (anteroposterior x transverse x craniocaudal, respectively) with a total volume of 383 cc.

The patient had an HbA1c of 8.6%. Screening for human immunodeficiency virus, hepatitis B and C was negative, and echocardiogram and chest radiograph were normal. Contrast-enhanced computed tomography of the abdomen revealed a multiloculated liver abscess 383 cc in size, periportal lymphadenopathy, and common bile duct calculus, as seen in Figure 1.

For treatment, pigtail insertion could not be done as liquefaction was inadequate. The patient was started on meropenem. Blood culture showed Escherichia coli growth, and he was started on amikacin based on culture sensitivity results. Diagnostic aspiration was done, in which 80 mL of greenish-yellow pus was aspirated under ultrasonogram guidance. Gram staining of the aspirate showed Gram-negative bacilli. The aspirate grew Enterobacter and Pseudomonas, which were sensitive to amikacin. The aspirated pus was sent for acid-fast stain, GeneXpert MTB (Cepheid, Sunnyvale, CA, USA), and mycobacterial culture. Adenosine deaminase (ADA) was 31.6 U/L. The acid-fast stain showed no acid-fast bacilli, but the GeneXpert MTB was positive. Once his liver function tests were normalized, the patient was started on a weight-based isoniazid, rifampicin, pyrazinamide, and ethambutol regimen.

The patient demonstrated clinical improvement, and serial ultrasonogram screening showed a gradual decrease in the size of the abscess. Mycobacterial culture grew acid-fast bacilli, confirming the diagnosis of tubercular abscess. For choledocholithiasis, endoscopic retrograde cholangiopancreatography was done, and a stent was placed, but the calculus could not be removed due to its position. He was discharged on anti-tubercular medication, and advised for stent and calculus removal on follow-up.

## Discussion

Liver abscesses are usually pyogenic, with approximately half of all visceral abscesses and 13% of intraabdominal abscesses being pyogenic [2]. Extrapulmonary TB accounts for more than 20% of the TB burden in India. Liver involvement is reported in 10%-15% of patients with extrapulmonary TB [3]. TB liver abscess is usually associated with a focus of infection in other organs such as the lungs or gastrointestinal tract. Liver involvement in TB can present in several forms [1], including diffuse with miliary TB or pulmonary TB [2], diffuse without another organ involvement [3], and focal lesions, multiple or solitary, which present as tuberculoma or abscess [4]. The prevalence of TB liver abscess is 0.34% [5]. Patients with TB liver abscess usually present with non-specific symptoms such as fever, loss of appetite, or right hypochondrial pain, with hepatomegaly being the most common finding on physical examination. Jaundice is an uncommon finding and might indicate biliary obstruction [3].

In our case, as the patient was immunocompromised, and TB is endemic in India, TB was part of our differential diagnosis. Radiological diagnosis in the diagnosis of hepatic TB has a low specificity [6]. Caseating granulomas on liver biopsy are diagnostic for TB but are not always present. If non-caseating granulomas are present, then an acid-fast test or culture is necessary for confirmation, but these are usually negative. A definite diagnosis can be made by demonstration of acid-fast bacilli in a specimen. Although the gold standard for diagnosis is mycobacterial culture, viable microorganisms must be present.

TB has a long incubation period of approximately 6-8 weeks. Cultures may be positive in 10% of cases and can be as high as 60% in miliary TB [7]. In a study by Diaz et al., 57% of tubercular hepatic granulomas showed a positive polymerase chain reaction (PCR) test [8]. In their study, 43 liver biopsy samples were taken, in which PCR had a sensitivity of 100% in the diagnosis of culture-positive Mycobacterium tuberculosis infection in lymph nodes, lungs, and liver. The sensitivity of PCR in the diagnosis of hepatic granuloma was 58%, and the specificity was 96% [8]. In studies done by Kim et al. [9] and Zeka et al. [10], GeneXpert MTB showed positivities of 47.7% and 65.5%, respectively, in smear-negative specimens. In a study done with 110 extrapulmonary TB samples, GeneXpert MTB showed a sensitivity of 87.25% and a specificity of 100% in culture-positive cases [11]. There are very few cases reported with GeneXpert MTB positivity in tubercular liver abscess. Agarwala et al. published a series of four cases of tubercular liver abscesses showing GeneXpert MTB positivity [12].

The preferred course of management is percutaneous drainage of the pus and anti-tubercular therapy [13]. Anti-tubercular therapy should be given for at least six months. Our case had a mixed infection with Enterobacter, Pseudomonas, and Mycobacterium tuberculosis. Although quite rare, mixed pyogenic and TB infections have been reported in the literature [14,15]. Singh et al. [16] reported a case of a hepatic abscess with co-infection of Proteus mirabilis and TB.

## Conclusions

Tubercular abscess is an uncommon manifestation of extrapulmonary TB. The clinical manifestations of the disease are non-specific. Tubercular abscess should be suspected in patients who have unresolved abscesses despite adequate treatment with antibiotics, especially in endemic areas.
